# Trends and Disparities in Adult Body Mass Index Across the 47 Prefectures of Japan, 1975–2018: A Bayesian Spatiotemporal Analysis of National Household Surveys

**DOI:** 10.3389/fpubh.2022.830578

**Published:** 2022-05-20

**Authors:** Nayu Ikeda, Tomoki Nakaya, James Bennett, Majid Ezzati, Nobuo Nishi

**Affiliations:** ^1^Section of Population Health Metrics, International Center for Nutrition and Information, National Institute of Health and Nutrition, National Institutes of Biomedical Innovation, Health and Nutrition, Tokyo, Japan; ^2^Graduate School of Environmental Studies, Tohoku University, Sendai, Japan; ^3^Department of Epidemiology and Biostatistics, School of Public Health, Faculty of Medicine, Imperial College London, London, United Kingdom; ^4^International Center for Nutrition and Information, National Institute of Health and Nutrition, National Institutes of Biomedical Innovation, Health and Nutrition, Tokyo, Japan

**Keywords:** body mass index, prefectures, Japan, Bayesian spatiotemporal model, National Health and Nutrition Survey, obesity

## Abstract

**Background:**

Among high-income countries, Japan has a low prevalence of obesity, but little is understood about subnational trends and variations in body mass index (BMI), largely owing to the lack of data from representative samples of prefectures. We aimed to examine long-term trends and distributions of adult BMI at the prefecture level in Japan from the late 1970s using a spatiotemporal model.

**Methods:**

We obtained cross-sectional data for 233,988 men and 261,086 women aged 20–79 years from the 44 annual National Health and Nutrition Surveys (NHNS) conducted during 1975–2018. We applied a Bayesian spatiotemporal model to estimate the annual time series of age-standardized and age-specific mean BMI by 20-year age group and sex for each of the 47 prefectures. We assessed socioeconomic inequalities in BMI across prefectures using the concentration index, according to population density.

**Results:**

In men, the age-standardized prefectural mean BMI ranged from 21.7 kg/m^2^ (95% credible interval, 21.6–21.9) to 23.1 kg/m^2^ (22.9–23.4) in 1975 and from 23.5 kg/m^2^ (23.3–23.7) to 24.8 kg/m^2^ (24.6–25.1) in 2018. In women, the age-standardized prefectural mean BMI ranged from 22.0 kg/m^2^ (21.9–22.2) to 23.4 kg/m^2^ (23.2–23.6) in 1975 and from 21.7 kg/m^2^ (21.6–22.0) to 23.5 kg/m^2^ (23.2–23.8) in 2018. Mean BMI was highest in the southernmost prefecture for most of the study period, followed by northeast prefectures. The increase in mean BMI was largest in southwest prefectures, which caught up with northeast prefectures over time. The concentration index was negative, indicating higher BMI in less-populated prefectures. Absolute values of the concentration index were greater in women than in men and increased over time.

**Conclusions:**

There were variations in adult mean BMI across prefectures, and geographic distributions changed over time. Further national and local efforts are needed to address the rising trend in mean BMI, particularly among men in rural prefectures, and socioeconomic inequalities among women. Bayesian hierarchical modeling is useful for reconstructing long-term spatiotemporal trends of mean BMI by integrating small-sized survey samples at the prefecture level in the NHNS.

## Introduction

The worldwide prevalence of adult overweight and obesity has been rising (1), and high body mass index (BMI) is considered to be one of the leading risk factors in the global burden of noncommunicable disease (2). Japan has some of the lowest mean BMI values among high-income and industrialized countries (1), as well as one of the highest life expectancies at birth in the world (3). In Japan, the mean BMI for men and women in 2016 was 23.7 kg/m^2^ and 21.8 kg/m^2^, respectively, whereas the respective global values were 24.5 kg/m^2^ and 24.8 kg/m^2^ (1). Understanding how body weight is controlled in the Japanese population could therefore help global public health professionals in developing effective policies and programs for obesity prevention.

Information on long-term trends and distributions of mean BMI among adults at the subnational level is essential for monitoring the progress of population-wide strategies to prevent obesity. In Japan, the National Health and Nutrition Survey (NHNS) is the main source of data on measured BMI in the adult population from the 1970s. The NHNS has been used to examine national trends in adult weight status (4–9) and the disease burden attributable to risk factors, including high BMI, at the subnational level (i.e., prefecture) in 2015 and 2019 (2, 10). However, it remains unknown how levels and distributions of adult BMI have changed across Japan's 47 prefectures over a long period because the NHNS was designed to be representative at the prefecture level only in 2012 and 2016 (11, 12).

In this study, we aimed to obtain stable estimates of annual mean BMI among adults across the 47 prefectures of Japan from 1975 to 2018. We applied the Bayesian spatiotemporal method to leverage the NHNS data at the prefecture level accumulated over the long term. We examined how spatial distributions of prefectural mean BMI had changed over four decades by sex and age. We also explored long-term trends in socioeconomic inequalities of prefectural mean BMI by sex.

## Materials and Methods

### Data Source and Study Participants

We used individual-level data from the National Nutrition Surveys between 1975 and 2002 and the National Health and Nutrition Surveys between 2003 and 2018 (henceforth both referred to as the NHNS) (13). The NHNS is an ongoing cross-sectional household interview and examination survey conducted annually by the Japanese government. We obtained government approval to use individual-level data according to the Statistics Act (14). No ethical review was required because use of the NHNS is exempt according to the Ethical Guidelines for Medical and Biological Research Involving Human Subjects (15).

Methodological details of survey sampling in the NHNS have been described elsewhere (16, 17). Briefly, the sampling frame was the list of all residential census enumeration areas stratified into 47 prefectures, with each census enumeration area consisting of approximately 50 households. The surveys used a stratified two-stage cluster sample design to obtain a nationally representative sample of the non-institutionalized Japanese population. Census enumeration areas were randomly drawn from each prefecture in the first sampling stage. Selected census enumeration areas were divided into unit blocks such that each block consisted of 20 to 30 households, and unit blocks were randomly sampled from each prefecture in the second stage. All individuals aged ≥1 year living in a private household in 300 sampled unit blocks were eligible for the survey. The expanded surveys in 2012 and 2016 used a stratified single-stage cluster sample design to obtain representative samples at the prefecture level. In these surveys, census enumeration areas were randomly drawn from each prefecture, and all residents aged ≥1 year in 475 selected census enumeration areas were eligible for the survey (11, 12).

All participants were asked to undergo physical examination. Standing height was measured to the nearest millimeter using a stadiometer and with participants wearing no shoes, and weight was measured to the nearest 0.1 kg with participants wearing light clothing. We calculated BMI as weight in kilograms divided by the square of height in meters.

We limited study participants to individuals aged 20 to 79 years. We obtained a sample of 495,074 survey participants (233,988 men and 261,086 women) from 1975 to 2018, after excluding 3,605 participants who were pregnant. We regarded BMI level <10 kg/m^2^ as implausible and recoded seven cases as having missing values accordingly. Of 495,074 participants, 110,984 (20.4%) had missing data for BMI. We filled in missing values of BMI and created five imputed datasets for analysis by predictive mean matching with one nearest neighbor for a single continuous variable. We applied this partially parametric method to have imputed BMI values within the range of observed data. We used sex, age in years, and prefecture as predictors in the imputation model and performed separate imputations by survey year. Supplementary Tables 1, 2 summarize the sample size and national mean BMI values estimated from multiple imputed datasets in each survey year.

### Statistical Analysis

In this analysis, we estimated mean BMI by age group and sex for all 47 prefectures of Japan from 1975 to 2018. We conducted all analyses separately by sex because levels and trends of the national mean BMI differed between men and women. We applied a Bayesian spatiotemporal model to obtain robust estimates of prefectural mean BMI from a small sample at the prefecture level (18). As data preparation, we used multiple imputed datasets to calculate point estimates and standard errors of the observed mean BMI by 20-year age groups (20–39, 40–59, and 60–79 years), sex, prefecture, and survey year. For the calculation of standard errors, we conducted mixed-effects linear regression of BMI on indicator variables for prefectures, accounting for the multistage survey sampling design that included stratification by prefecture and clustering by census enumeration areas.

We formulated the Bayesian spatiotemporal model to incorporate features of prefectural mean BMI in relation to age, prefecture, and survey year. In the model, we assumed that an observed prefectural mean BMI (Y_apt_) in age group *a* (= 1, 2, 3), prefecture *p* (= 1,...,47), and year *t* (= 1,...,44) follows a normal distribution,


$$\begin{array}{l} Y_{\text{atp}}\sim N\left(\mu_{\text{atp}},\;\sigma^{2}se_{\text{atp}}^{2}\right). \end{array}$$

The parameter μ_atp_ signifies the expected value of prefectural mean BMI and σ^2^se${}_{\text{atp}}^{2}$ signifies the variance of prediction error weighted by squared standard errors of the observed mean BMI specific to age, year and prefecture, se_atp_, reflecting the sample size in the survey. We weighted each data point by squared standard errors such that data points with smaller standard errors had a greater influence on the estimated prefectural mean BMI. We specified Y_atp_ as a linear function of time,

Y_atp_ = (α_0_ + β_0_ × t) + (α_1a_ + β_1a_ × t) + (α_2p_ + β_2p_ × t) + (α_3ap_ + β_3ap_ × t) + γ_1at_ + γ_2pt_

where α_0_ and β_0_ are the common intercept and trend, respectively, across age groups and prefectures.

The age-specific intercept (α_1a_) and slope (β_1a_) quantify the deviation in age group *a* from α_0_ in level and β_0_ in trend, respectively. To ensure smoothness over adjacent age groups, we used a first-order random walk prior on α_1a_ and β_1a_, holding α_11_ and β_11_ for the youngest group to 0. The first-order random walk takes the general form of α_1a_ ~ N(α_1a−1_, $\sigma_{\text{α}1}^{2}$).

The prefecture-specific intercept (α_2p_) and slope (β_2p_) measure the deviation in prefecture *p* from α_0_ in level and β_0_ in trend, respectively. We modeled α_2p_ and β_2p_ using the Besag, York, and Mollié (BYM) model, which allows mean BMI in each prefecture to be estimated using its own data and those of its neighboring prefectures. In the BYM model, information is shared both locally between neighboring prefectures through spatially structured random effects with a conditional autoregressive prior, and globally among all prefectures through spatially unstructured (independent and identically distributed) Gaussian random effects (19). We imposed the spatial structure of the conditional autoregressive prior through the adjacency matrix that specified the geographic proximity among the 47 prefectures. A map of prefectures is provided with information on population density in Supplementary Figure 1. We considered a pair of prefectures to be neighbors if they shared a land border or were connected by a bridge or tunnel across the sea. We joined Okinawa, the southernmost prefecture, to the nearest prefecture (Kagoshima) based on ferry connections from island to island. There was no change in boundaries among prefectures during the study period. Maps of the spatially smoothed random effects component of the intercepts and slopes are shown by sex in Supplementary Figure 2.

The age-prefecture interaction terms α_3ap_ and β_3ap_ account for age-specific deviations in intercepts and slopes, respectively, in prefecture *p* from those of other prefectures. The α_3ap_ and β_3ap_ were assumed to be independent and identically distributed Gaussian random effects. The model included first-order random walks over time to allow for nonlinearity from the average linear trend for each age group (γ_1at_) and each prefecture (γ_2pt_). We used weakly informative priors such that inference in parameters was driven by data. All standard-deviation parameters had σ ~ U(0,2) priors. For the global intercept (α_0_) and slope (β_0_), we used N(0, 100,000).

We fitted the Bayesian spatiotemporal model with the Markov chain Monte Carlo algorithm in WinBugs 1.4.3 (20). For each analysis by sex, we ran the model with two chains until convergence was reached. We visually monitored convergence of the chains using trace plots. Convergence was reached with 5,000 iterations, and we ran a further 5,000 iterations on each chain to collect 10,000 postburn in samples for inference from the posterior distributions of parameters. We averaged these samples to yield point estimates of prefectural mean BMI and reported 95% credible intervals as the 2.5th and the 97.5th percentiles of the 10,000 samples.

Using estimated prefectural mean BMI, we computed the concentration index to examine socioeconomic inequality in BMI among prefectures. The concentration index is a relative measure of inequality that indicates the extent to which a health indicator is concentrated according to socioeconomic status (21). The concentration index ranges from −1 to 1 and equals zero when there is no inequality. It takes a negative value when a health variable is concentrated at lower socioeconomic levels. As a socioeconomic variable of prefecture, we used population density (total population per square kilometer of total land area) by age group, sex, prefecture, and year. Population density is a stable measure of urbanization of prefectures over the four decades. To generate this variable, we divided the age- and sex-specific total population by total land area in each prefecture and each year obtained from the System of Social and Demographic Statistics (22). As a health variable, we generated the total BMI per square kilometer of total land area calculated as the product of mean BMI and population density. We sorted prefectures in ascending order of population density and calculated cumulative proportions of population density and total BMI in each prefecture. We computed the concentration index as twice the sum of the difference between the cumulative proportions of population density and total BMI across prefectures (21).

We used R version 3.6.0 (www.r-project.org) for data preparation and Stata version 15 (StataCorp LP, College Station, TX, USA) for data preparation and analysis of the concentration index. For age-standardization, we obtained the total population, by 20-year age groups, from the 2010 Population Census of Japan (23).

## Results

### Annual Changes in the Distribution of Prefectural Mean BMI

**Figure 1:**
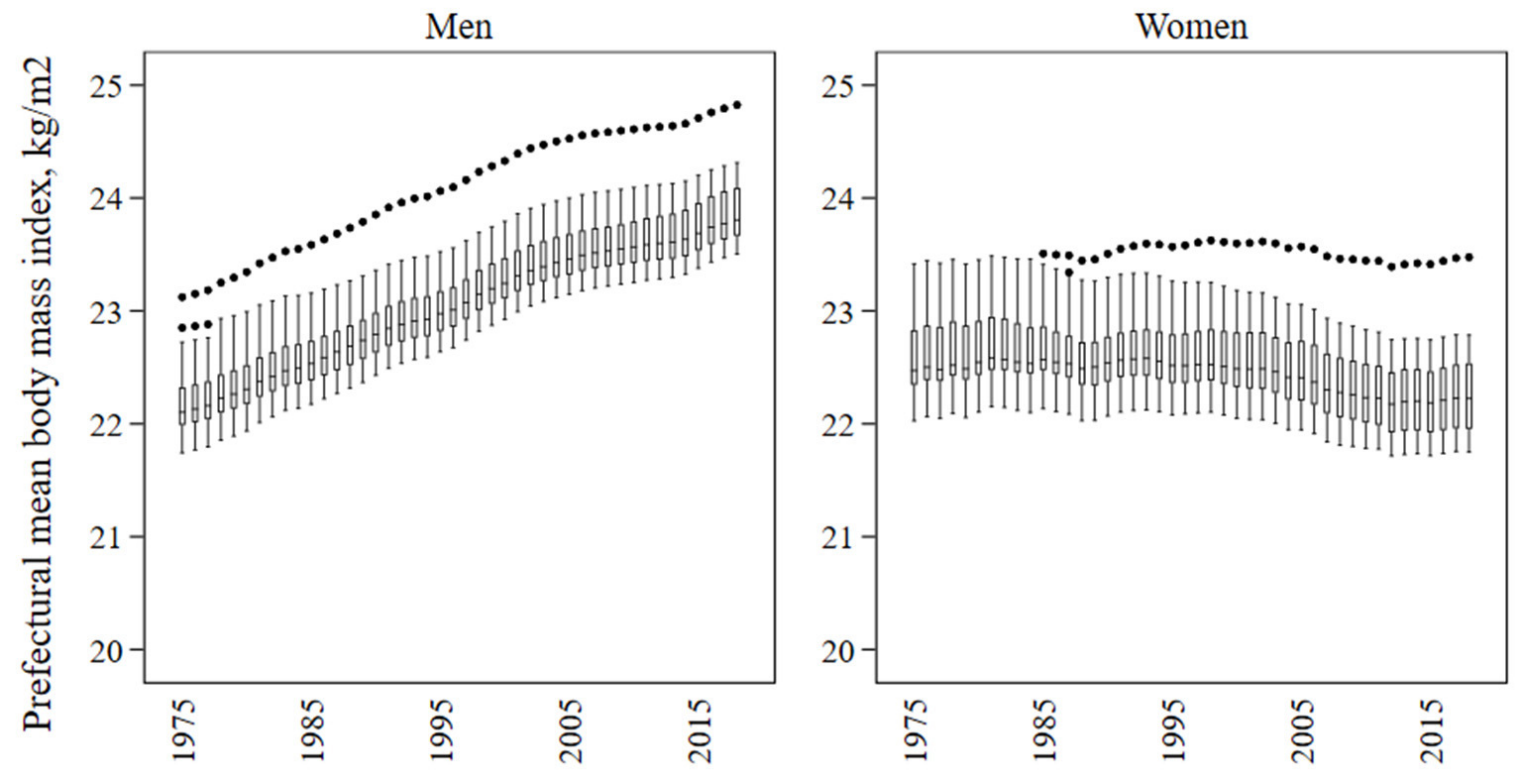
Distribution of the age-standardized mean body mass index (kg/m^2^) across the 47 prefectures of Japan in each year from 1975 to 2018. The box shows the 25th, 50th, and 75th percentiles. The lower adjacent line indicates minimum values or values less than the first quartile minus 1.5 times the interquartile range. The upper adjacent line indicates maximum values or values greater than the third quartile plus 1.5 times the interquartile range. Points show values below the lower adjacent value or above the upper adjacent value.

Figure 1 demonstrates annual changes in the distribution of age-standardized mean BMI values estimated by the Bayesian spatiotemporal model across the 47 prefectures between 1975 and 2018. Among men, the whole distribution of the age-standardized prefectural mean BMI consistently shifted upward during the study period, and similar trends were found for the distributions of prefectural mean BMI by age group (Supplementary Figure 3). Among women, the distribution of age-standardized prefectural mean BMI remained almost constant until starting to gradually shift downward in the 2000s (Figure 1). Trends varied among age groups in women (Supplementary Figure 3). The distribution of the prefectural mean BMI started to shift downward during the 1980s among young and middle-aged women whereas in older women, it continued to move upward until starting to shift downward in the early 2000s. The age-standardized mean BMI was substantially higher in Okinawa than in other prefectures across survey years for men and from the late 1980s for women (Figure 1).

### Spatiotemporal Changes in Prefectural Mean BMI

**Figure 2:**
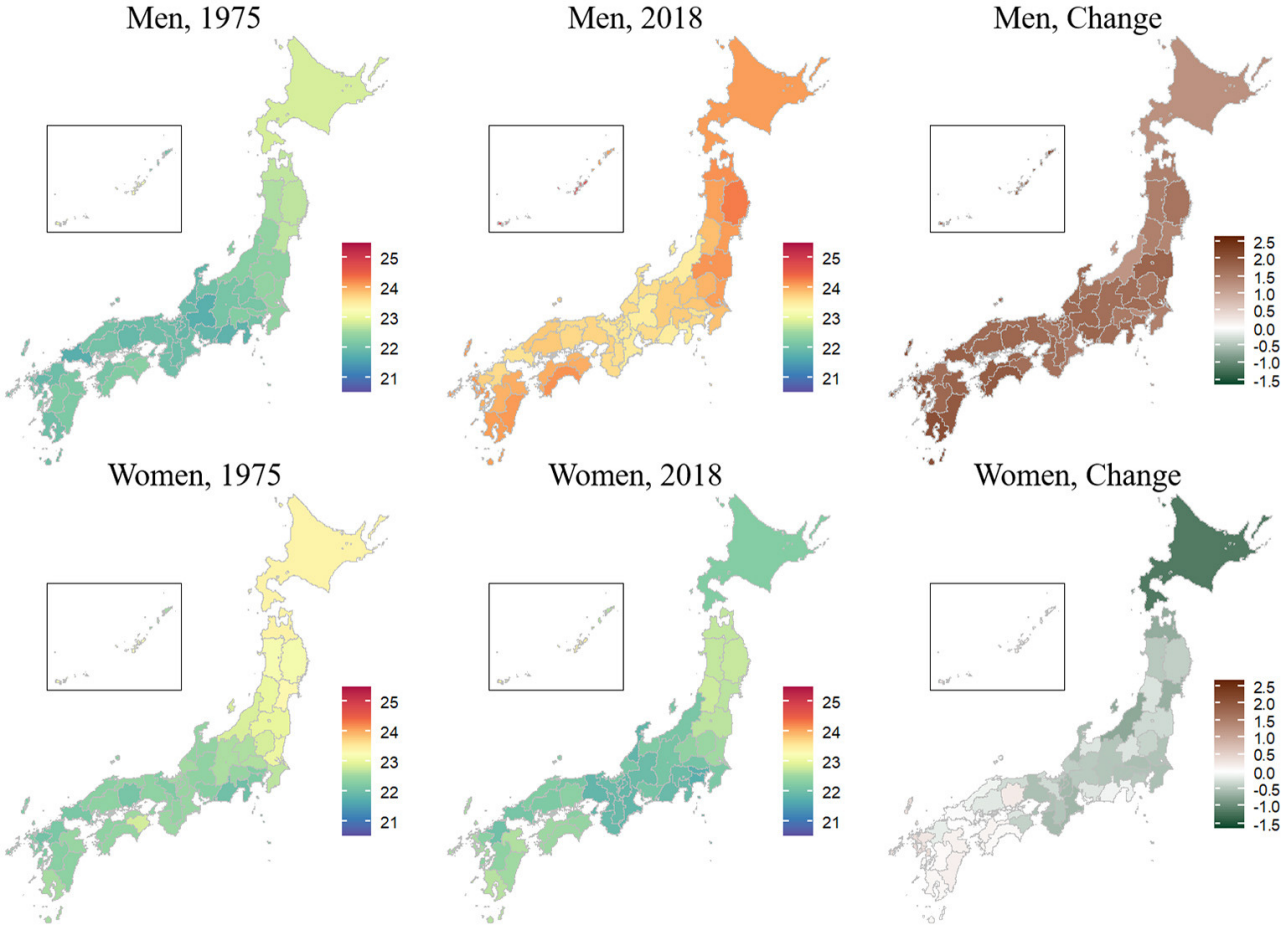
Geography of the age-standardized prefectural mean body mass index (kg/m^2^) in 1975 and 2018 and its changes among adults aged 20–79 years in the 47 prefectures of Japan, by sex. The prefectural mean body mass index was estimated using a Bayesian spatiotemporal model. The insets are enlarged views of Okinawa.

Maps in Figure 2 show changes in spatial distributions of age-standardized prefectural mean BMI. The age-standardized mean BMI in 1975 was higher in Hokkaido, Okinawa, and northeast prefectures than in other prefectures for both sexes. The geographic distribution of the age-standardized prefectural mean BMI had changed by 2018. The age-standardized mean BMI was highest in Okinawa, followed by those in northeast and southwest prefectures for both sexes. The age-standardized mean BMI increased more in southwest prefectures than in other prefectures for both sexes over time. The decrease in age-standardized mean BMI for women was largest in Hokkaido (1.2 kg/m^2^ [95% credible interval, 0.8–1.5]).

Similar spatiotemporal trends were found for age-specific prefectural mean BMI values across all sex and age groups (Supplementary Figures 4, 5). The increase in mean BMI between 1975 and 2018 was largest for men aged 60–79 years in southwest prefectures. The decrease in mean BMI was largest for women aged 40–59 years in Hokkaido.

### Changes in Socioeconomic Inequality for BMI Across Prefectures

**Figure 3:**
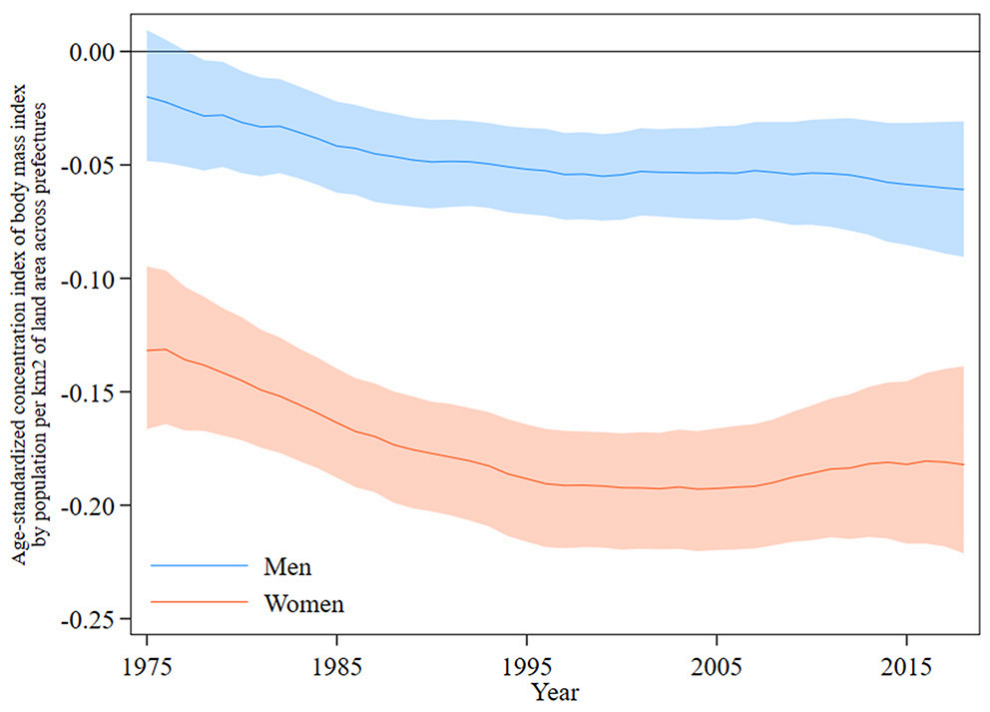
Concentration index for the total body mass index across the 47 prefectures of Japan in each year from 1975 to 2018, by sex. The height of the shaded area shows the 95% credible interval.

Figure 3 shows trends in the age-standardized concentration index for total BMI according to total population per square kilometer of total land area across prefectures. The concentration index was negative for both sexes and was lower in women than in men throughout the study period. The concentration index in 1975 was −0.02 (95% credible interval, −0.05 to 0.01) for men and −0.13 (−0.17 to −0.09) for women. The concentration index in men gradually decreased over time to −0.06 (−0.09 to −0.03) in 2018. In women, it decreased relatively rapidly to −0.19 (−0.22 to −0.17) in 2004 and thereafter remained stable at −0.18 (−0.22 to −0.14) in 2018.

## Discussion

Using NHNS data and the Bayesian spatiotemporal method, we obtained stable annual estimates of mean BMI among adults across the 47 prefectures of Japan over four decades. Our study is novel in that we revealed long-term changes in geographic and socioeconomic variations of prefectural mean BMI. Such information on subnational variations in mean BMI is important for the evaluation of national health promotion strategies, but this has been unclear using NHNS samples directly, which are not representative at the prefecture level. The estimation method is valid (18) and has been used to examine long-term trends and distributions of BMI indicators across countries worldwide (24–26). This method has also been applied to improve district-level population health estimates from national surveys in various countries, such as the under-five mortality in Ghana (27), child nutrition in South Africa (28), and life expectancy in the United Kingdom (29, 30).

Our results confirmed that long-term trends in prefectural mean BMI values were different between men and women. Prefectural mean BMI in men increased steadily across all age groups throughout the study period. Prefectural mean BMI in young and middle-aged women decreased during the 1980s; in older women, these values increased until starting to decrease in the early 2000s. These overall trends of prefectural mean BMI were consistent with previous findings on national trends estimated using the NHNS for individuals aged 20 years and over between 1976 and 1995 (5), women aged 15–29 years between 1976 and 2000 (6), and individuals aged 65 years and over between 1973 and 2016 (8). Thus, our model was able to reflect national trends in the estimation of prefectural mean BMI.

Our results suggest that mean BMI has been relatively high in northeast prefectures and Okinawa throughout the study period, which might be attributable to lifestyles developed under climatic and historical environments. Around the late 1970s, a relatively high mean BMI was mainly observed in northeast prefectures. This result might partly reflect prolonged exposure to unfavorable lifestyles in a cold climate, such as physical inactivity and a diet high in salt. Historically, dietary salt intake has been high in northeast regions to maintain body temperature and preserve food for storage (31–33). A positive association of dietary salt intake with BMI was found in a multi-country study (34). Moreover, the trends in overweight and obesity were already obvious among men living in Okinawa during the late 1970s. One explanation for the distinctly high mean BMI in Okinawa at that time might be lifestyles under American occupation between 1945 and 1972. In fact, animal fat intake from imported meat products increased rapidly in Okinawa during the 1960s, as compared with Western countries (35). Our finding of high mean BMI in Okinawa agrees with that of a previous longitudinal analysis based on self-reported height and weight in the 1990s and early 2000s (36). Okinawa was once ranked first in longevity but has shown relatively poor performance in recent years, which may be partly attributable to the distinctive trends in adult BMI.

We found that the geographic distribution of prefectural mean BMI changed over time, mainly because southwest prefectures caught up with northeast prefectures. In recent years, prefectural mean BMI has been relatively high toward the northeast and southwest rural regions of Japan. One explanation for this geographic change in mean BMI might be a decrease in dietary salt intake across the country over time (37). The suppressing effect of reduced dietary salt consumption on the rise in mean BMI might be relatively large in northeast prefectures. Another possible explanation might be decreased physical activity, particularly in less-populated prefectures, within increased motorization and use of privately owned cars rather than public transport. Previous studies in Japan show a positive association between the size of a municipality and number of walking steps per day (38) and an inverse association between walkability of a residential neighborhood and BMI (39, 40).

Another key finding of this study was that socioeconomic inequalities in overweight and obesity have increased over time in Japan. The concentration index of BMI was negative, as expected, reflecting an inverse relationship between mean BMI and population density across prefectures. Widening socioeconomic inequalities might be partly attributable to the relatively rapid increase in mean BMI in less-populated southwest prefectures. Moreover, socioeconomic inequalities were markedly larger in women than in men. This difference between men and women might partly reflect the increased concentration of adult women who are employed in the Tokyo metropolitan area (41), where mean BMI is rather low for women. A previous study in Japan reported that young adult women living in metropolitan areas are more likely to want to be thin than those living in towns (42).

From a public health perspective, a series of national health promotion campaigns have been implemented since the late 1970s to improve lifestyles in the Japanese population (43). Health Japan 21 (the second term), underway during fiscal years 2013 to 2023, is a campaign that has set numerous targets that include reducing health inequalities and increasing the proportion of people who can maintain a healthy weight (44). To achieve these targets, local governments, such as prefectures and municipalities, have developed their own basic plans under the Health Promotion Act (45). The NHNS in 2012 and 2016 were conducted using expanded samples to obtain prefecture-level estimates for subnational evaluation of Health Japan 21 (the second term) (11, 12). Despite these efforts, mean BMI in some prefectures was still inconsistent between the two surveys. For example, mean BMI among middle-aged women in Kyoto was the seventh highest in 2012, at 23.2 kg/m^2^; it was the second lowest in 2016, at 22.0 kg/m^2^. The lack of consistency and comparability in estimates might be partly owing to methodological issues such as survey sampling and implementation in each prefecture and each year. The ranking of prefectural mean BMI reported in the NHNS would therefore be somewhat misleading for the assessment of local public health promotion programs. The Bayesian spatiotemporal method applied in this study would compensate for such shortcomings by integrating small-sized survey samples to reconstruct long-term trends of prefecture-level mean BMI.

Our findings and implications are relevant to global public health policies for the prevention of obesity. The prevalence of overweight and obesity among OECD countries in 2019 was lowest at 27% in Japan, followed by 34% in South Korea, while it exceeded 70% in Chile, Mexico, and the United States (46). The prevalence of overweight and obesity has been rising in all OECD countries, especially it has increased by 15% or more between 2009 and 2019 in Chile, Mexico, and Turkey. Even in Japan, a high-income country with a low obesity prevalence, there are subnational variations in BMI and geographic distributions and socioeconomic inequalities have changed over time. Mean BMI has been steadily increasing across prefectures particularly for men, and the increase was larger in less-populated prefectures toward the northeast and southwest rural regions of the country. These results partly support a previous finding of a persistently higher BMI in rural areas in high-income and industrialized countries (25).

Our study has several methodological limitations that should be addressed in future research. First, we did not examine the prevalence of underweight, overweight, and obesity with a different probability distribution from that of mean BMI. Long-term trends of the prevalence of these BMI categories at the prefecture level should be assessed as a next step in spatiotemporal analyses based on the NHNS. Second, it would be challenging to obtain prefecture-level estimates on other important cardiometabolic risk factors such as blood pressure, blood glucose, and serum cholesterol because samples of these items are even smaller than those of body height and weight in the NHNS (33). A possible solution for this challenge might be modification of the model by adding hierarchical structures of prefectures. Finally, the Bayesian spatiotemporal method is still too difficult to be used in regular monitoring of prefecture-level BMI in the NHNS. The development of simplified models is necessary to introduce the estimation method in routine survey practice.

In conclusion, there were variations in adult mean BMI across prefectures, and geographic distributions changed over time. Further national and local efforts are needed to address the rising trend in mean BMI, particularly among men in less-populated prefectures in the northeast and southwest rural regions, and socioeconomic inequalities among women. Understanding body weight status at the subnational level is essential to formulate effective national and local strategies for ensuring health and well-being in countries worldwide that are aiming for sustainable development. Global efforts should be continued to strengthen coherent actions across sectors in establishing food systems that deliver a balanced and healthy diet at an affordable price for all individuals, as well as residential environments that promote daily walking and physical activity. Bayesian spatiotemporal modeling is a promising approach for using existing national surveys in the assessment of health system performance to control obesity at the subnational level.

## Data Availability Statement

The data analyzed in this study is subject to the following licenses/restrictions: Statistics Act. Requests to access these datasets should be directed to NI, ikedan@nibiohn.go.jp.

## Ethics Statement

Ethical review and approval was not required for the study on human participants in accordance with the local legislation and institutional requirements. Written informed consent for participation was not required for this study in accordance with the national legislation and the institutional requirements.

## Author Contributions

NI designed the study, created data applications for the government, conducted statistical analyses, and drafted the article. TN developed the adjacent matrix and provided input on spatial analyses in Japan. JB helped draft the statistical codes and provided input on the Bayesian spatiotemporal model. ME provided guidance for spatiotemporal analyses at the subnational level from the perspective of global health. NN supervised the study. TN, JB, ME, and NN interpreted the results and critically revised the article for important intellectual content. All authors contributed to the article and approved the submitted version.

## Funding

This study was supported by the Japan Society for the Promotion of Science (grant no. 18H03063).

## Conflict of Interest

The authors declare that the research was conducted in the absence of any commercial or financial relationships that could be construed as a potential conflict of interest.

## Publisher's Note

All claims expressed in this article are solely those of the authors and do not necessarily represent those of their affiliated organizations, or those of the publisher, the editors and the reviewers. Any product that may be evaluated in this article, or claim that may be made by its manufacturer, is not guaranteed or endorsed by the publisher.

## Supplementary Material

Supplementary Figure 1 Map of the 47 prefectures of Japan, colored by total population per square kilometer of total land area in 2010 from the system of social and demographic statistics.

Supplementary Figure 2 Maps of the spatially smoothed random effects component of the intercepts and slopes by sex. The variance of these spatially structured components accounted for between 51% and 99% of the variance of intercepts and slopes for both men and women.

Supplementary Figure 3 Distribution of the age-specific mean body mass index (kg/m^2^) across the 47 prefectures of Japan in each year from 1975 to 2018. The box shows the 25th, 50th, and 75th percentiles. The lower adjacent line indicates minimum values or values less than the first quartile minus 1.5 times the interquartile range. The upper adjacent line indicates maximum values or values greater than the third quartile plus 1.5 times the interquartile range. Points show values below the lower adjacent value or above the upper adjacent value.

Supplementary Figure 4 Geography of the age-specific prefectural mean body mass index (kg/m^2^) in 1975 and 2018 and its changes among men aged 20–79 years in the 47 prefectures of Japan. The prefectural mean body mass index was estimated using a Bayesian spatiotemporal model. The insets are enlarged views of Okinawa.

Supplementary Figure 5 Geography of the age-specific prefectural mean body mass index (kg/m^2^) in 1975 and 2018 and its changes among women aged 20–79 years in the 47 prefectures of Japan. The prefectural mean body mass index was estimated using a Bayesian spatiotemporal model. The insets are enlarged views of Okinawa.

Supplementary Table 1 Sample size and mean body mass index estimated from multiple imputed datasets by age group and survey year in male participants aged 20–79 years.

Supplementary Table 2 Sample size and mean body mass index estimated from multiple imputed datasets by age group and survey year in female participants aged 20–79 years.
