# Supplementary figures and images for: Trends and Disparities in Adult Body Mass Index Across the 47 Prefectures of Japan, 1975–2018: A Bayesian Spatiotemporal Analysis of National Household Surveys
Source: Front Public Health. 2022 May 20;10:830578. doi: 10.3389/fpubh.2022.830578 (PMC9163402; doi:10.3389/fpubh.2022.830578)

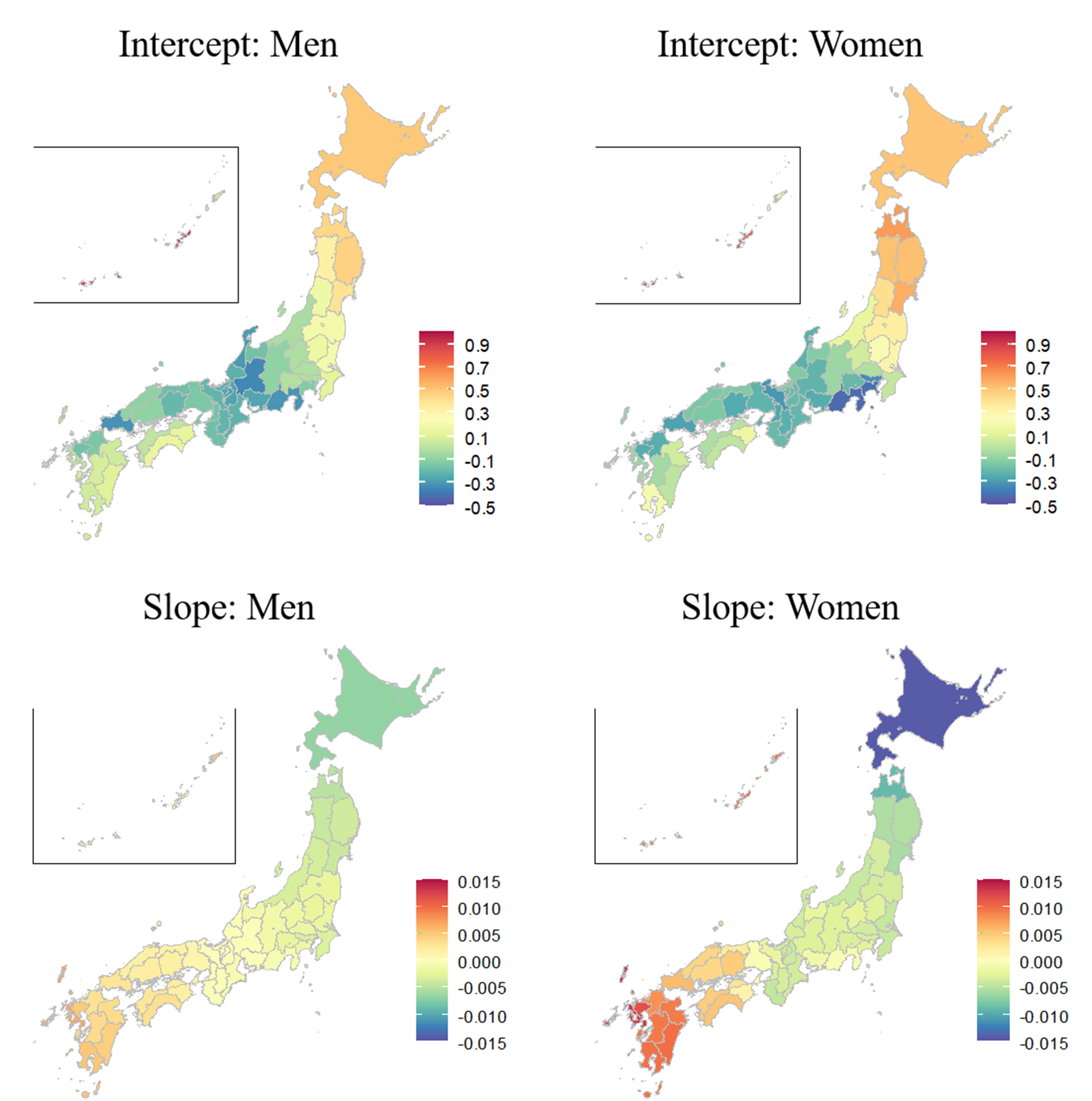

Supplement: Supplementary Figure 1 — Map of the 47 prefectures of Japan, colored by total population per square kilometer of total land area in 2010 from the system of social and demographic statistics. [file Image_1.TIF]

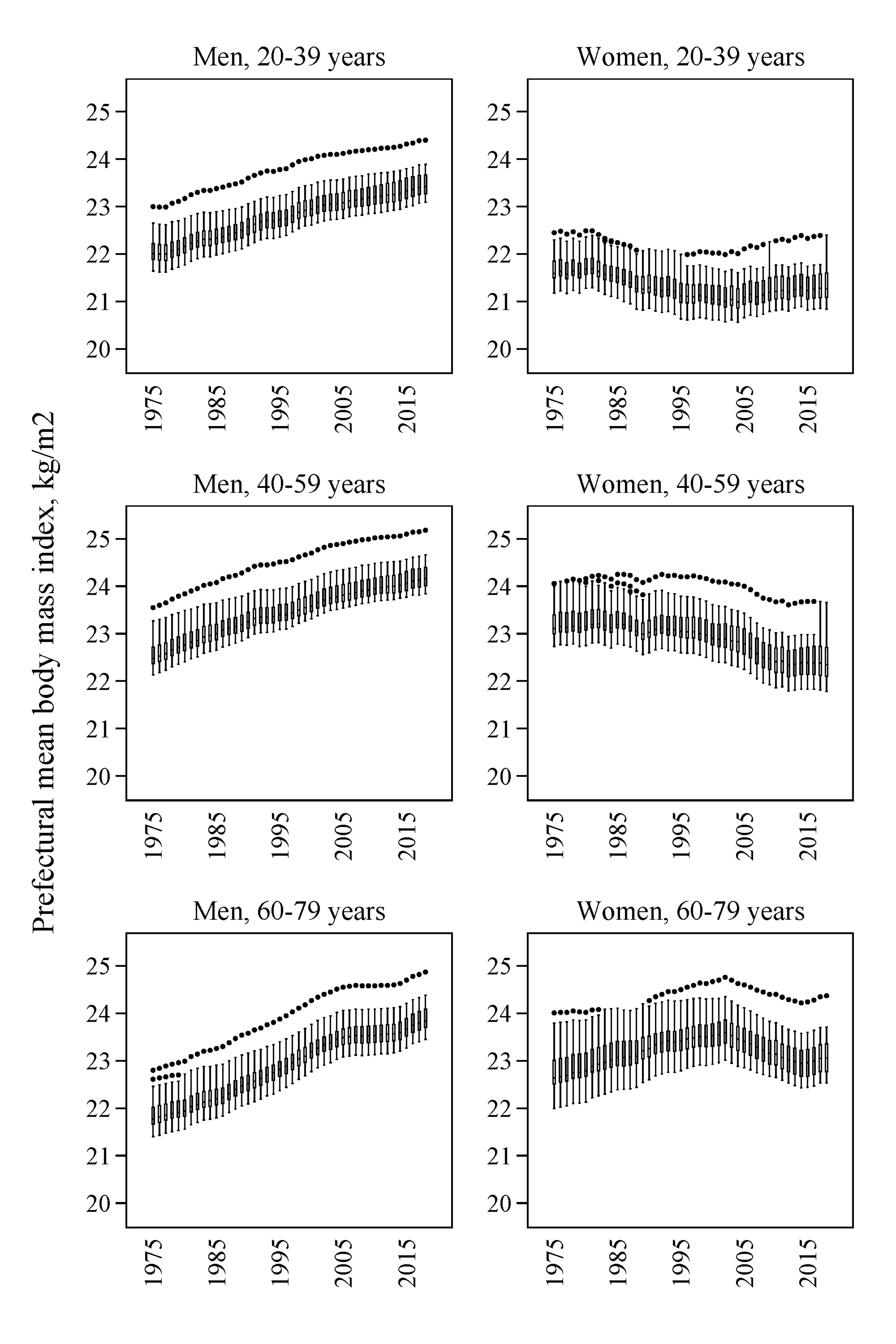

Supplement: Supplementary Figure 2 — Maps of the spatially smoothed random effects component of the intercepts and slopes by sex. The variance of these spatially structured components accounted for between 51% and 99% of the variance of intercepts and slopes for both men and women. [file Image_2.TIFF]

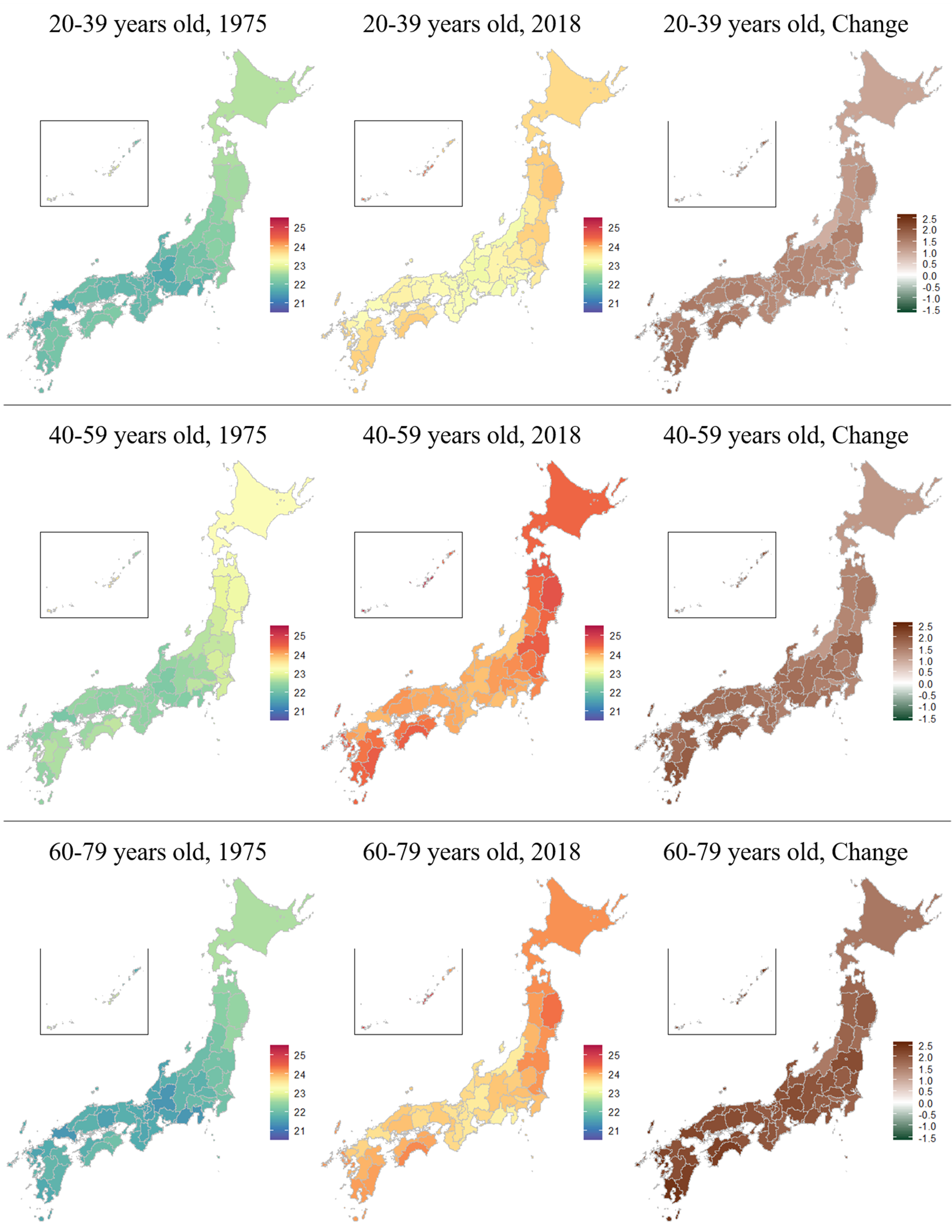

Supplement: Supplementary Figure 3 — Distribution of the age-specific mean body mass index (kg/m2) across the 47 prefectures of Japan in each year from 1975 to 2018. The box shows the 25th, 50th, and 75th percentiles. The lower adjacent line indicates minimum values or values less than the first quartile minus 1.5 times the interquartile range. The upper adjacent line indicates maximum values or values greater than the third quartile plus 1.5 times the interquartile range. Points show values below the lower adjacent value or above the upper adjacent value. [file Image_3.TIF]

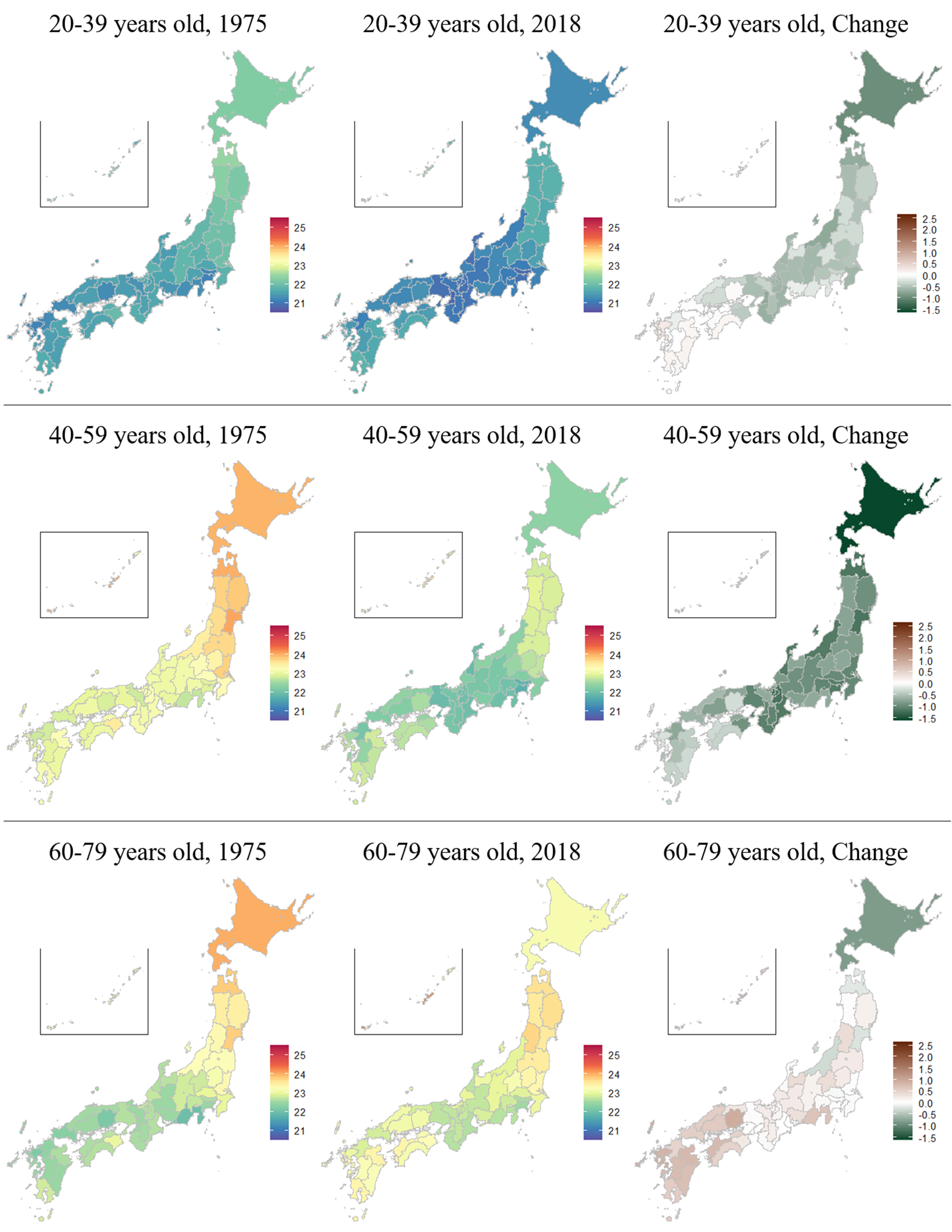

Supplement: Supplementary Figure 4 — Geography of the age-specific prefectural mean body mass index (kg/m2) in 1975 and 2018 and its changes among men aged 20–79 years in the 47 prefectures of Japan. The prefectural mean body mass index was estimated using a Bayesian spatiotemporal model. The insets are enlarged views of Okinawa. [file Image_4.TIF]

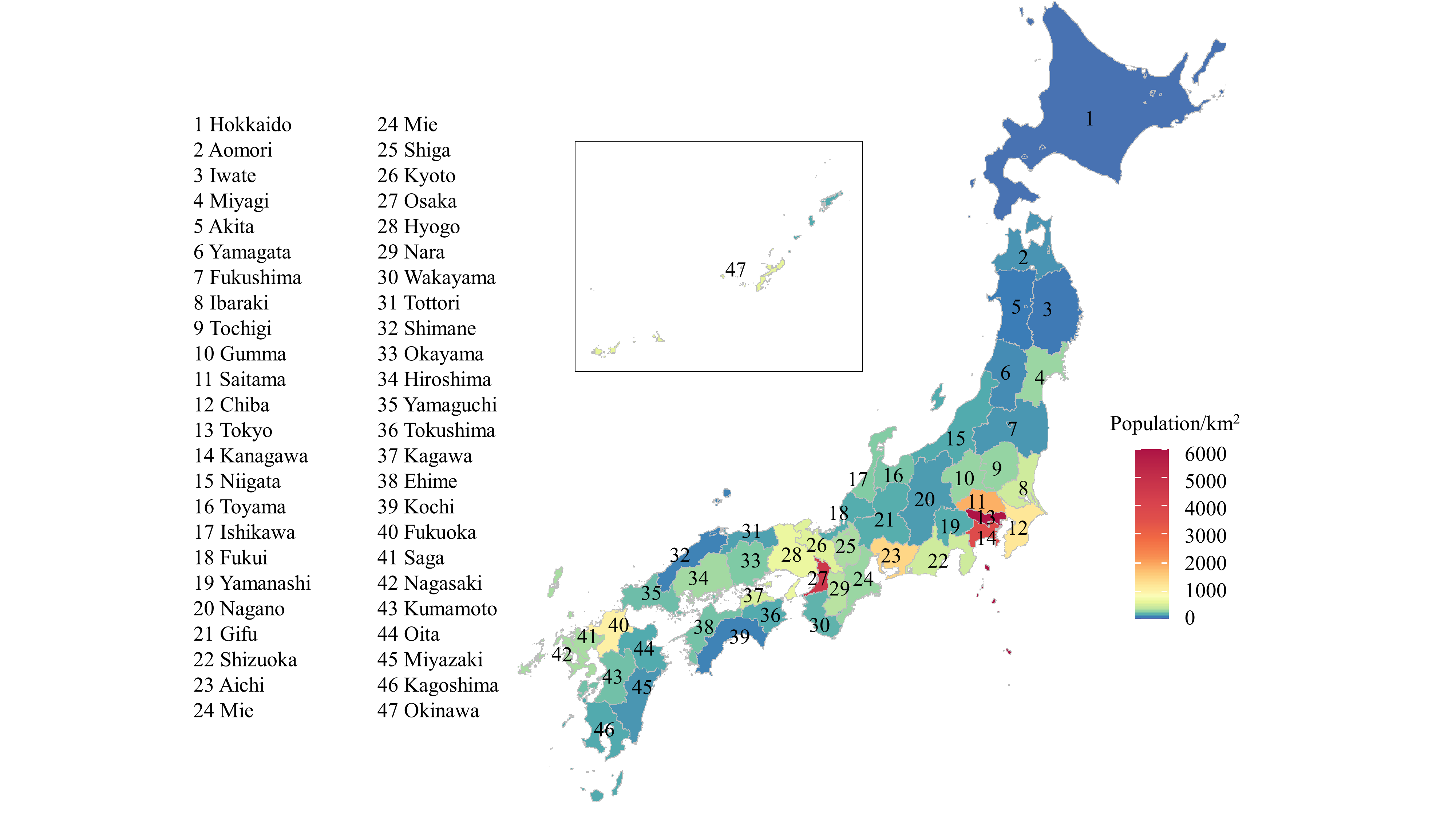

Supplement: Supplementary Figure 5 — Geography of the age-specific prefectural mean body mass index (kg/m2) in 1975 and 2018 and its changes among women aged 20–79 years in the 47 prefectures of Japan. The prefectural mean body mass index was estimated using a Bayesian spatiotemporal model. The insets are enlarged views of Okinawa. [file Image_5.TIF]
