# Supplementary material for: Trends and Disparities in Adult Body Mass Index Across the 47 Prefectures of Japan, 1975–2018: A Bayesian Spatiotemporal Analysis of National Household Surveys
Source: Front Public Health. 2022 May 20;10:830578. doi: 10.3389/fpubh.2022.830578 (PMC9163402; doi:10.3389/fpubh.2022.830578)
Supplement: Supplementary Table 1 — Sample size and mean body mass index estimated from multiple imputed datasets by age group and survey year in male participants aged 20–79 years. [file Table_1.DOCX]

Appendix Table 1. Sample size and mean body mass index estimated from multiple imputed datasets by age group and survey year in male participants aged 20–79 years

| Year | Sample size | | Mean BMI estimated from multiple imputed datasets, kg/m^2^ | | | | | |
| --- | --- | --- | --- | --- | --- | --- | --- | --- |
|  | Total | BMI imputed | 20–39 years | | 40–59 years | | 60–79 years | |
| 1975 | 6,769 | 2,347 | 22.1 | (22.0, 22.3) | 22.6 | (22.5, 22.7) | 21.9 | (21.6, 22.1) |
| 1976 | 8,144 | 2,839 | 22.0 | (21.9, 22.1) | 22.6 | (22.5, 22.8) | 21.9 | (21.7, 22.1) |
| 1977 | 6,495 | 2,284 | 21.9 | (21.8, 22.0) | 22.6 | (22.4, 22.7) | 21.9 | (21.7, 22.2) |
| 1978 | 6,770 | 2,207 | 22.2 | (22.0, 22.3) | 22.8 | (22.6, 22.9) | 22.0 | (21.8, 22.2) |
| 1979 | 6,665 | 1,817 | 22.1 | (22.0, 22.2) | 22.8 | (22.6, 22.9) | 22.0 | (21.8, 22.2) |
| 1980 | 7,262 | 1,710 | 22.2 | (22.0, 22.3) | 22.9 | (22.7, 23.0) | 21.8 | (21.6, 22.0) |
| 1981 | 6,575 | 2,647 | 22.2 | (22.1, 22.4) | 22.9 | (22.7, 23.0) | 22.3 | (22.1, 22.5) |
| 1982 | 6,582 | 2,029 | 22.4 | (22.2, 22.5) | 22.9 | (22.8, 23.0) | 22.2 | (22.0, 22.4) |
| 1983 | 6,645 | 2,279 | 22.4 | (22.3, 22.6) | 23.1 | (22.9, 23.2) | 22.4 | (22.2, 22.6) |
| 1984 | 6,431 | 2,124 | 22.3 | (22.1, 22.4) | 23.0 | (22.9, 23.2) | 22.2 | (22.0, 22.3) |
| 1985 | 6,506 | 1,403 | 22.4 | (22.2, 22.5) | 22.9 | (22.8, 23.1) | 22.3 | (22.1, 22.5) |
| 1986 | 6,485 | 1,499 | 22.4 | (22.2, 22.6) | 23.2 | (23.1, 23.3) | 22.1 | (22.0, 22.3) |
| 1987 | 6,377 | 1,921 | 22.4 | (22.3, 22.6) | 23.1 | (23.0, 23.2) | 22.4 | (22.2, 22.6) |
| 1988 | 5,940 | 1,072 | 22.4 | (22.3, 22.6) | 23.2 | (23.0, 23.3) | 22.5 | (22.3, 22.7) |
| 1989 | 5,500 | 1,446 | 22.4 | (22.3, 22.5) | 23.2 | (23.0, 23.3) | 22.6 | (22.4, 22.8) |
| 1990 | 6,027 | 1,707 | 22.6 | (22.5, 22.8) | 23.3 | (23.2, 23.4) | 22.5 | (22.3, 22.6) |
| 1991 | 5,610 | 1,231 | 22.6 | (22.4, 22.9) | 23.4 | (23.3, 23.5) | 22.7 | (22.5, 22.8) |
| 1992 | 5,298 | 1,179 | 22.7 | (22.6, 22.9) | 23.4 | (23.3, 23.6) | 22.6 | (22.4, 22.8) |
| 1993 | 5,262 | 1,110 | 22.9 | (22.7, 23.0) | 23.3 | (23.2, 23.5) | 22.8 | (22.6, 23.0) |
| 1994 | 4,969 | 918 | 22.5 | (22.4, 22.7) | 23.3 | (23.2, 23.5) | 22.6 | (22.4, 22.8) |
| 1995 | 4,816 | 778 | 22.8 | (22.6, 22.9) | 23.5 | (23.3, 23.7) | 22.8 | (22.6, 23.1) |
| 1996 | 4,904 | 846 | 22.6 | (22.4, 22.8) | 23.4 | (23.2, 23.5) | 22.8 | (22.7, 23.0) |
| 1997 | 4,618 | 712 | 22.8 | (22.6, 22.9) | 23.4 | (23.2, 23.5) | 22.9 | (22.8, 23.1) |
| 1998 | 4,998 | 831 | 23.0 | (22.9, 23.2) | 23.6 | (23.4, 23.7) | 23.1 | (23.0, 23.3) |
| 1999 | 4,515 | 1,073 | 23.0 | (22.8, 23.2) | 23.6 | (23.4, 23.8) | 23.0 | (22.9, 23.2) |
| 2000 | 4,355 | 718 | 22.8 | (22.6, 23.0) | 23.5 | (23.3, 23.6) | 23.3 | (23.1, 23.5) |
| 2001 | 4,330 | 851 | 23.1 | (22.9, 23.3) | 23.7 | (23.6, 23.9) | 23.4 | (23.2, 23.5) |
| 2002 | 4,103 | 855 | 23.1 | (22.9, 23.3) | 23.8 | (23.6, 24.0) | 23.4 | (23.2, 23.6) |
| 2003 | 4,186 | 994 | 23.1 | (22.8, 23.4) | 23.8 | (23.7, 24.0) | 23.3 | (23.1, 23.5) |
| 2004 | 3,365 | 747 | 23.0 | (22.8, 23.2) | 23.8 | (23.6, 24.0) | 23.6 | (23.4, 23.8) |
| 2005 | 3,421 | 904 | 22.9 | (22.6, 23.2) | 23.8 | (23.6, 24.0) | 23.6 | (23.5, 23.8) |
| 2006 | 3,509 | 751 | 23.3 | (23.0, 23.6) | 23.9 | (23.7, 24.1) | 23.5 | (23.4, 23.7) |
| 2007 | 3,432 | 657 | 23.1 | (22.9, 23.4) | 23.8 | (23.6, 24.0) | 23.7 | (23.5, 23.9) |
| 2008 | 3,569 | 764 | 23.0 | (22.7, 23.3) | 24.0 | (23.8, 24.2) | 23.5 | (23.3, 23.7) |
| 2009 | 3,495 | 748 | 23.3 | (23.0, 23.6) | 23.9 | (23.7, 24.1) | 23.6 | (23.4, 23.7) |
| 2010 | 3,492 | 948 | 23.1 | (22.9, 23.3) | 24.1 | (23.9, 24.3) | 23.5 | (23.4, 23.7) |
| 2011 | 3,073 | 689 | 23.4 | (23.1, 23.7) | 24.0 | (23.8, 24.2) | 23.6 | (23.4, 23.8) |
| 2012 | 13,210 | 4,207 | 23.3 | (23.1, 23.5) | 24.1 | (24.0, 24.2) | 23.6 | (23.5, 23.7) |
| 2013 | 3,125 | 587 | 23.1 | (22.8, 23.5) | 23.9 | (23.7, 24.2) | 23.5 | (23.3, 23.7) |
| 2014 | 3,315 | 801 | 23.0 | (22.6, 23.4) | 23.9 | (23.7, 24.1) | 23.5 | (23.3, 23.6) |
| 2015 | 3,054 | 794 | 23.6 | (23.2, 24.0) | 24.0 | (23.7, 24.2) | 23.6 | (23.5, 23.8) |
| 2016 | 11,014 | 3,314 | 23.4 | (23.2, 23.6) | 24.2 | (24.1, 24.4) | 23.8 | (23.7, 23.9) |
| 2017 | 2,950 | 833 | 23.7 | (23.3, 24.1) | 24.0 | (23.8, 24.2) | 23.8 | (23.6, 24.0) |
| 2018 | 2,827 | 636 | 23.2 | (22.8, 23.6) | 24.1 | (23.9, 24.4) | 23.9 | (23.7, 24.1) |

BMI, body mass index. Values in parentheses indicate lower and upper bounds of 95% confidence intervals accounting for the complex survey sampling design that included stratification by prefecture and clustering by census enumeration units in each survey year.

Appendix Table 2. Sample size and mean body mass index estimated from multiple imputed datasets by age group and survey year in female participants aged 20–79 years

| Year | Sample size | | Mean BMI estimated from multiple imputed datasets, kg/m^2^ | | | | | |
| --- | --- | --- | --- | --- | --- | --- | --- | --- |
|  | Total | BMI imputed | 20–39 years | | 40–59 years | | 60–79 years | |
| 1975 | 7,634 | 1,273 | 21.5 | (21.4, 21.7) | 23.1 | (23.0, 23.3) | 22.7 | (22.5, 23.0) |
| 1976 | 9,147 | 1,623 | 21.7 | (21.6, 21.8) | 23.3 | (23.1, 23.4) | 22.8 | (22.6, 23.0) |
| 1977 | 7,208 | 1,195 | 21.5 | (21.4, 21.6) | 23.2 | (23.1, 23.3) | 22.8 | (22.6, 23.0) |
| 1978 | 7,386 | 1,042 | 21.7 | (21.6, 21.8) | 23.3 | (23.1, 23.4) | 23.0 | (22.8, 23.2) |
| 1979 | 7,464 | 840 | 21.5 | (21.4, 21.6) | 23.1 | (23.0, 23.3) | 22.9 | (22.6, 23.1) |
| 1980 | 7,998 | 993 | 21.7 | (21.6, 21.9) | 23.2 | (23.1, 23.4) | 22.8 | (22.6, 23.0) |
| 1981 | 7,320 | 1,561 | 21.7 | (21.6, 21.8) | 23.3 | (23.1, 23.4) | 23.0 | (22.8, 23.2) |
| 1982 | 7,314 | 917 | 21.7 | (21.5, 21.8) | 23.3 | (23.2, 23.5) | 23.0 | (22.9, 23.2) |
| 1983 | 7,399 | 1,120 | 21.5 | (21.4, 21.7) | 23.2 | (23.1, 23.3) | 23.1 | (22.9, 23.3) |
| 1984 | 7,103 | 1,100 | 21.5 | (21.4, 21.6) | 23.1 | (22.9, 23.2) | 23.1 | (22.9, 23.3) |
| 1985 | 7,152 | 622 | 21.5 | (21.4, 21.6) | 23.3 | (23.2, 23.4) | 23.2 | (23.0, 23.4) |
| 1986 | 7,143 | 727 | 21.4 | (21.3, 21.5) | 23.2 | (23.1, 23.4) | 23.1 | (22.9, 23.3) |
| 1987 | 7,208 | 1,073 | 21.4 | (21.3, 21.6) | 23.2 | (23.1, 23.4) | 23.1 | (23.0, 23.3) |
| 1988 | 6,641 | 603 | 21.2 | (21.1, 21.3) | 23.1 | (22.9, 23.2) | 23.0 | (22.8, 23.2) |
| 1989 | 6,161 | 887 | 21.2 | (21.1, 21.4) | 22.9 | (22.8, 23.0) | 23.3 | (23.1, 23.5) |
| 1990 | 6,711 | 1,123 | 21.3 | (21.2, 21.5) | 23.0 | (22.9, 23.2) | 23.3 | (23.1, 23.4) |
| 1991 | 6,255 | 771 | 21.2 | (21.1, 21.4) | 23.1 | (23.0, 23.3) | 23.4 | (23.2, 23.6) |
| 1992 | 5,957 | 807 | 21.1 | (21.0, 21.3) | 23.2 | (23.0, 23.3) | 23.4 | (23.2, 23.6) |
| 1993 | 5,916 | 774 | 21.3 | (21.1, 21.5) | 23.0 | (22.9, 23.2) | 23.5 | (23.3, 23.7) |
| 1994 | 5,549 | 600 | 21.2 | (21.0, 21.3) | 23.1 | (23.0, 23.2) | 23.3 | (23.1, 23.5) |
| 1995 | 5,466 | 510 | 21.0 | (20.8, 21.1) | 23.0 | (22.9, 23.2) | 23.4 | (23.2, 23.6) |
| 1996 | 5,499 | 593 | 21.0 | (20.9, 21.2) | 23.0 | (22.8, 23.2) | 23.5 | (23.3, 23.7) |
| 1997 | 5,314 | 490 | 21.0 | (20.8, 21.1) | 23.0 | (22.9, 23.2) | 23.4 | (23.2, 23.7) |
| 1998 | 5,530 | 575 | 21.1 | (21.0, 21.3) | 23.0 | (22.8, 23.2) | 23.6 | (23.4, 23.8) |
| 1999 | 5,140 | 798 | 21.1 | (20.9, 21.2) | 23.0 | (22.8, 23.2) | 23.4 | (23.2, 23.6) |
| 2000 | 4,851 | 541 | 21.0 | (20.8, 21.2) | 22.8 | (22.7, 23.0) | 23.5 | (23.3, 23.7) |
| 2001 | 4,971 | 602 | 21.0 | (20.9, 21.2) | 22.8 | (22.6, 23.0) | 23.5 | (23.3, 23.6) |
| 2002 | 4,600 | 652 | 20.8 | (20.6, 21.0) | 22.9 | (22.7, 23.1) | 23.8 | (23.6, 24.0) |
| 2003 | 4,608 | 647 | 21.2 | (21.0, 21.4) | 22.8 | (22.6, 23.0) | 23.5 | (23.4, 23.7) |
| 2004 | 3,817 | 604 | 20.7 | (20.5, 20.9) | 22.8 | (22.6, 23.0) | 23.3 | (23.1, 23.5) |
| 2005 | 3,837 | 796 | 21.2 | (20.9, 21.4) | 22.8 | (22.6, 23.0) | 23.4 | (23.2, 23.6) |
| 2006 | 3,923 | 576 | 21.3 | (21.1, 21.5) | 22.7 | (22.5, 22.9) | 23.3 | (23.1, 23.5) |
| 2007 | 3,847 | 537 | 20.9 | (20.7, 21.1) | 22.5 | (22.3, 22.7) | 23.2 | (23.0, 23.4) |
| 2008 | 4,044 | 657 | 21.1 | (20.9, 21.4) | 22.4 | (22.2, 22.6) | 23.2 | (23.0, 23.4) |
| 2009 | 3,968 | 656 | 21.2 | (21.0, 21.5) | 22.5 | (22.3, 22.7) | 23.0 | (22.8, 23.2) |
| 2010 | 3,850 | 753 | 21.2 | (20.9, 21.4) | 22.2 | (22.0, 22.4) | 23.2 | (23.0, 23.4) |
| 2011 | 3,462 | 627 | 21.3 | (21.1, 21.6) | 22.5 | (22.3, 22.8) | 23.0 | (22.8, 23.2) |
| 2012 | 14,556 | 3,538 | 21.2 | (21.1, 21.4) | 22.4 | (22.2, 22.5) | 23.1 | (23.0, 23.2) |
| 2013 | 3,459 | 541 | 21.3 | (21.0, 21.6) | 22.3 | (22.1, 22.6) | 22.9 | (22.7, 23.1) |
| 2014 | 3,665 | 749 | 21.7 | (21.3, 22.0) | 22.4 | (22.2, 22.6) | 22.8 | (22.6, 23.0) |
| 2015 | 3,450 | 690 | 21.0 | (20.8, 21.2) | 22.3 | (22.1, 22.6) | 22.8 | (22.6, 23.0) |
| 2016 | 12,316 | 3,067 | 21.4 | (21.2, 21.6) | 22.5 | (22.4, 22.6) | 23.0 | (22.9, 23.1) |
| 2017 | 3,129 | 694 | 21.3 | (21.0, 21.7) | 22.3 | (22.1, 22.6) | 23.1 | (22.9, 23.3) |
| 2018 | 3,118 | 633 | 21.3 | (21.0, 21.6) | 22.3 | (22.0, 22.6) | 23.0 | (22.8, 23.2) |

BMI, body mass index. Pregnant participants were excluded from the analysis. Values in parentheses indicate lower and upper bounds of 95% confidence intervals accounting for the complex survey sampling design that included stratification by prefecture and clustering by census enumeration units in each survey year.
